# The selective biotin tagging and thermolysin proteolysis of chloroplast outer envelope proteins reveals information on protein topology and association into complexes

**DOI:** 10.3389/fpls.2014.00203

**Published:** 2014-05-16

**Authors:** Hélène Hardré, Lauriane Kuhn, Catherine Albrieux, Juliette Jouhet, Morgane Michaud, Daphné Seigneurin-Berny, Denis Falconet, Maryse A. Block, Eric Maréchal

**Affiliations:** ^1^Laboratoire de Physiologie Cellulaire et Végétale, UMR 5168 CNRS-CEA-INRA-Université Grenoble Alpes, iRTSVCEA Grenoble, Grenoble, France; ^2^Laboratoire de Biologie à Grande Echelle, iRTSVCEA Grenoble, Grenoble, France

**Keywords:** chloroplast envelope, outer envelope protein, membrane protein complexes, biotinylation, thermolysin

## Abstract

The understanding of chloroplast function requires the precise localization of proteins in each of its sub-compartments. High-sensitivity mass spectrometry has allowed the inventory of proteins in thylakoid, stroma, and envelope fractions. Concerning membrane association, proteins can be either integral or peripheral or even soluble proteins bound transiently to a membrane complex. We sought a method providing information at the surface of the outer envelope membrane (OEM), based on specific tagging with biotin or proteolysis using thermolysin, a non-membrane permeable protease. To evaluate this method, envelope, thylakoid, and stroma proteins were separated by two-dimensional electrophoresis and analyzed by immunostaining and mass spectrometry. A short selection of proteins associated to the chloroplast envelope fraction was checked after superficial treatments of intact chloroplasts. We showed that this method could allow the characterization of OEM embedded proteins facing the cytosol, as well as peripheral and soluble proteins associated *via* tight or lose interactions. Some stromal proteins were associated with biotinylated spots and analyzes are still needed to determine whether polypeptides were tagged prior import or if they co-migrated with OEM proteins. This method also suggests that some proteins associated with the inner envelope membrane (IEM) might need the integrity of a trans-envelope (IEM–OEM) protein complex (e.g., division ring-forming components) or at least an intact OEM partner. Following this evaluation, proteomic analyzes should be refined and the putative role of inter-membrane space components stabilizing trans-envelope complexes demonstrated. For future comprehensive studies, perspectives include the dynamic analyses of OEM proteins and IEM–OEM complexes in various physiological contexts and using virtually any other purified membrane organelle.

## Introduction

The chloroplast is a specific organelle inside plant and algal cells, involved in numerous biochemical functions, including photosynthesis. This organelle derives from an ancestral cyanobacterium following an endosymbiotic event. Its membrane compartmentation is undoubtedly one of the most complex found in eukaryotic cells. In higher plants, chloroplasts are delineated by an envelope made of two membranes, the outer and the inner envelope membrane, OEM and IEM, respectively. In the stroma, additional membrane sacks, the thylakoids, provide an extensive surface for light capture and photosynthetic energy conversion. Liquid phase compartments comprise the inter-membrane space between the OEM and IEM, the stroma, and the lumen of thylakoids. Understanding the mechanisms that orchestrate the chloroplast biogenesis, differentiation and function, requires a fine sub-organellar localization of the chloroplast components. The characterization of cytosolic components associated with the envelope surface is further needed to understand the functional integration of the organelle within the rest of the cell.

The vast majority of plastid proteins are encoded by nuclear genes. Protein precursors are synthesized within the cytosol with an *N*-terminal chloroplastic transit peptide (Ctp). For major chloroplast precursors (small subunit of the Rubisco, the light harvesting complex β, subunits of the oxygen evolving complex and the ATPase γ-subunit), the Ctp is phosphorylated in the cytosol, stimulating the association of a hetero-oligomeric complex, the guidance complex, with a 14-3-3 dimer, a cytosolic heat shock protein HSP70/Com70, and possibly several other components (Pontier et al., 2007; Li and Chiu, 2010; Lee et al., 2013). The Ctp subsequently binds to the general import machinery, the TOC/TIC translocon, which directs precursors across the envelope membranes (Li and Chiu, 2010). After import, the Ctp is cleaved by a stromal processing peptidase (for review, Li and Chiu, 2010). This process is sufficient to address the majority of stromal and IEM proteins. Nevertheless, some Ctp-less precursors were shown to be addressed to the IEM (Tranel and Keegstra, 1996; Miras et al., 2002, 2007). With the noticeable exception of Toc75 harboring a bipartite transit peptide (Tranel et al., 1995; Tranel and Keegstra, 1996), most OEM proteins have no cleavable addressing signal and follow independent import systems (Li and Chiu, 2010). Thus, although some general processes appear to target the bulk of proteins inside chloroplast sub-compartments, none is universal, and even though Ctp features are useful for computational predictive methods, no bioinformatic tool allows a prediction of a protein targeting to the OEM with a high confidence level (Schleiff et al., 2003). Accuracy of predictions being imperfect, assessment of sub-organellar proteomes had to be determined experimentally.

Proteomic analyzes have clarified this point with an unprecedented level of precision. The strategy consisted in purifying intact chloroplasts, fractionate sub-organellar compartments (envelope, stroma, thylakoids), resolve protein sub-fractions by 1-D or 2-D PAGE and identify individual proteins based on mass spectrometry analyses. This was performed first using spinach chloroplast leaves as a convenient starting material yielding high amounts of pure chloroplasts and, with improvement of analytical sensitivity, with *Arabidopsis* leaves, benefiting of all the genomic information made available for this model. A difficulty was that 2D-PAGE resolution was unsuccessful in the recovery of trans-membrane proteins and the yield of less hydrophobic proteins decreased with loaded protein amounts. The main constraint limiting the analysis of integral proteins is due to hydrophobic polypeptides, which cannot be resolved by isoelectric focusing (IEF) and electrophoresis, even under stringent denaturing conditions. To circumvent this problem, the most hydrophobic envelope proteins were selectively extracted using organic solvents. The resulting extract could be resolved by 1-D SDS-PAGE, and proteins identified by mass spectrometry methods (Seigneurin-Berny et al., 1999; Ferro et al., 2000; Rolland et al., 2003). Owing to the selective solubility fractionation, integral envelope proteins were inventoried for the first time and miss-localizations corrected. Having performed and validated the subcellular localization of envelope protein markers by immunostaining and chloroplast visualization of GFP-protein fusions, 2D-PAGE based proteomic studies have served as references for comprehensive and highly sensitive proteome characterization of envelope sub-comparments in *Arabidopsis* (Rolland et al., 2006; Salvi et al., 2008; Joyard et al., 2010), which results have been made accessible via the AT_CHLORO database (Ferro et al., 2010) (http://www.grenoble.prabi.fr/at_chloro/).

A major challenge is to get access to topological information (what is inward and outward a given membrane) and how protein complexes get associated. In the present paper, we proceeded stepwise. For the convenience of pure organelle pre-treatments, we used spinach chloroplasts as a model. We used conditions allowing the resolution of envelope-associated proteins by 2-D PAGE, a procedure also efficient for the resolution of thylakoid and stroma proteins. Because no gold standard has been defined for 2-D PAGE analysis of chloroplast envelope proteins, the quality of the 2-D profiles we obtained was assessed by immunostaining with antibodies raised against envelope, thylakoid and stroma protein markers. Further protein identification was based on mass spectrometry analyses. Proteins exposed at the surface of chloroplasts were sought by complementary methods, i.e., selective superficial biotin-tagging and thermolysin-proteolysis. In addition to known envelope membrane proteins, our analyses of intact or tagged/shaved envelope membranes, showed that *soluble* proteins were also detected at the periphery of the envelope, sometimes associated to stable trans-envelope complexes. From this characterization, several processes were shown to be structurally associated to the envelope: a channeling of stromal protein maturation, a dynamic assembly and structural stability of some stromal and trans-envelope complexes and several important steps of stromal RNA editing. Our work introduces therefore a “tag and shave” strategy as a possible approach to characterize peripheral membrane proteins of a membrane bound organelle, bringing topological clues concerning the sub-organellar localization of proteins and their possible involvement in large functional complexes connecting sub-compartments. This technical development was performed on spinach leaves so as to provide abundant organellar material, and future directions using more accurate plant models are discussed.

## Materials and methods

### Isolation of purified intact spinach chloroplast and preparation of spinach chloroplast subfractions

All operations were carried out at 0–5°C. Spinach leaves were obtained freshly from the market and kept overnight in the dark at 4°C so as to reduce the starch content (Joyard et al., 1982). Crude chloroplasts were isolated from 3 kg of spinach (*Spinacia oleracea* L.) leaves. Envelope, stroma, and thylakoid subfractions from the chloroplasts were purified as described previously (Joyard et al., 1982). All manipulations were performed at 4°C. In brief, deveined spinach leaves were homogenized in 2 L of sucrose 0.33 M, Na-pyrophosphate 30 mM, Bovine serum albumin 1 g·L^−1^, pH 7.8, for 2 s in a 4-L Waring Blendor and a crude chloroplast pellet was obtained from the leaf homogenate. The pellet was washed in sucrose 0.33 M, MOPS 10 mM, pH 7.8. To avoid contamination by other membrane organelles and swollen thylakoid membranes, the chloroplast preparation was purified further by isopycnic centrifugation on a Percoll (Pharmacia) gradient (40% Percoll, 50 mL and 80% Percoll, 20 mL in washing buffer; 5000 *g*, 20 min). Intact chloroplasts were collected at the interface between the 40 and 80% Percoll cushions. At this stage, thermolysin treatment or biotin tagging was performed as described below. Envelope, thylakoids, and stroma were prepared from purified, intact chloroplasts after swelling in a hypotonic medium (MOPS 10 mM, MgCl_2_ 4 mM, EDTA 5 mM, pH 7.8 in presence of protease inhibitors) followed by ultra-centrifugation through a step sucrose gradient (sucrose 0.6 M, 10 mL and 0.93 mM, 12 mL in MOPS 10 mM, MgCl_2_ 4 mM, EDTA 5 mM, pH 7.8 in presence of protease inhibitors; 72,000 *g*, 1 h). The swelling medium as well as the different sucrose layers contained the following protease inhibitors: EDTA, 5 mM, phenylmethylsulfonylfluoride, 1 mM; E-aminocaproic acid, 5 mM; and benzamidine-HCI, 1 mM. The yield of envelope membranes was 2–3 mg of protein/kg of spinach leaves.

### Protease treatment of isolated intact spinach chloroplast

To study polypeptides localized on the external face of the OEM, intact spinach chloroplast were treated with thermolysin from *Bacillus thermoproteolyticus* (Boehringer Mannheim, Germany). Protease treatments were carried out on ice, under light conditions and using intact chloroplasts at 1 mg·mL^−1^ of chlorophyll in buffer T containing 100 μM thermolysin, 0.33 M saccharose, 20 mM MOPS pH 7.8, 1 mM CaCl_2_. The reaction was terminated after 1 h with EGTA (10 mM). Treated chloroplasts were layered on a Percoll gradient as described above, in presence of EDTA (10 mM) and centrifuged at 5000 *g* for 20 min to obtain intact plastids. Intact treated chloroplasts were used to purify envelope, stroma, and thylakoid sub-fractions as described above (Joyard et al., 1982), in presence of EDTA (5 mM) and of a cocktail of protease inhibitors. Samples that were not treated with protease (mock) went through the same procedure except that buffer T was deprived of thermolysin.

### Biotinylation of isolated intact spinach chloroplast

To study polypeptides of the OEM, intact spinach chloroplasts were superficially labeled with the hydrophobic biotinylation reagent 6-((6-((biotinoyl)amino)hexanoyl)amino)hexanoic acid, succinimidyl ester (biotin-XX,SE; Molecular Probes). Biotinylation reaction was performed during 15 min at 4°C. Chlorophyll content of intact chloroplasts was measured as described (Arnon, 1949). Chloroplast labeling reactions were carried out on ice using intact chloroplasts at 5 mg·mL^−1^ of chlorophyll in buffer B containing 20 μM of biotin-XX,SE, 0.33 M saccharose, 50 mM sodium bicarbonate pH 8.3, 0.1% (v/v) Dimethyl Sulfoxide (DMSO). The biotinylation reaction was terminated after 15 min with hydroxylamine 5 mM. Biotinylated chloroplasts were layered on a Percoll gradient in 0.33 M saccharose, 50 mM MOPS pH 7.8, and centrifuged at 5000 *g* for 20 min to obtain intact plastids as described above. intact treated chloroplasts were suspended in 0.33 M saccharose, 50 mM MOPS pH 7.8, in presence of EDTA (5 mM) and protease inhibitors and envelope, stroma, and thylakoid sub-fractions were purified as described above. Samples that were not biotinylated (mock) went through the same procedure except that buffer B was deprived of biotin-XX,SE.

### One-dimensional polyacrylamide gel electrophoresis (1-D SDS-PAGE)

Proteins prepared from intact or biotinylated spinach chloroplast sub-fractions (20 μg proteins, determined using the BCA protein assay kit, BioVision, and bovine serum albumin as a standard) were separated by SDS-PAGE (11% polyacrylamide gel) according to standard procedures. Separated proteins were either electro-transferred to nitrocellulose membranes or stained with Coomassie brilliant blue G- 250.

### Two-dimensional polyacrylamide gel electrophoresis (2-D PAGE)

All reagents and materials were obtained from Bio-Rad unless indicated. Polypeptides of the chloroplast sub-fractions (stroma, thylakoid, and envelope membranes) were analyzed by 2-D PAGE. Each sub-fraction (200 μg proteins) was solubilized in 250 μl of a rehydratation buffer containing 8 M urea, 2 M thiourea, 4% (w/v) CHAPS, 100 mM dithiothreitol (DTT), and 0.2% (v/v) Bio-lytes (0.1% of pH 4–6 + 0.1% of pH 5–7 or 0.2% of pH 3–10). After 30-min incubation at 20°C, the solubilized proteins were used for passive hydration of linear immobilized pH gradient (IPG) strips (7 cm; pH 4–7 or pH 3–10). Then, the 7 cm IPG strips were subjected to the following IEF program using a Bio-Rad Protean IEF System: constant voltage at 50 V for 4 h; constant voltage at 250 V for 2 h; linear increase from 250 to 4000 V over 9 h and constant voltage set at 4000 V for a total of 25 kV·h. The current was limited (50 μA per strip), and the running temperature was set at 20°C. The strips were stored at −20°C until used for second dimension. To solubilize proteins focused during the first dimension run, IPG strips were equilibrated for 20 min in equilibration buffer 1 containing 6 M urea, 20% (w/v) glycerol, 2% (w/v) SDS, 375 mM Tris-HCl, pH 8.8, 130 mM DTT, and for 40 min in equilibration buffer 2 containing 6 M urea, 20% (w/v) glycerol, 2% (v/v) SDS, 375 mM Tris-HCl, pH 8.8, 135 mM iodoacetamide. After equilibration, IPG strips were loaded on top of a 13% acrylamide gel and fixed with molten agarose solution to ensure good contact between gel and strip. Twelve gels were cast under identical conditions within multi-casting chambers. A BioRad Dodeca cell was used to ensure that gels were run under the same electrical conditions. Electrophoresis was performed at 20°C in the following buffer: 25 mM Tris-HCl, 192 mM Glycine and 2% (v/v) SDS for 1 h at 25 V followed by 2 h at 100 V. Separated proteins were either electro-transferred to nitrocellulose membranes or stained with Coomassie brilliant blue G- 250.

### Immunoblotting studies of proteins

Electro-transfer of proteins separated by SDS-PAGE or 2-D PAGE on nitrocellulose membranes (Hybond-ECL, Amersham, Pharmacia Biotech) was carried out using standard procedures. Western blot analyses were achieved using 300 mM NaCl, 10 mM Tris-HCl, pH 7.5, containing non-fat dried milk (50 g·L^−1^) as blocking buffer. All antibodies were from rabbit sera. Polyclonal antibodies raised against spinach OEP10 and OEP24 [anti-OEP10 and anti-OEP24, (Joyard et al., 1982)], which are specific marker of the OEM, spinach IEP37 [anti-IEP37, (Joyard et al., 1982)], a major IEM polypeptide, spinach MGDG synthase 1 [anti-MGD1, (Awai et al., 2001)], a minor IEM polypeptide, and polyclonal antibodies against the recombinant *Arabidopsis* protein ceQORH [anti-ceQORH, (Miras et al., 2002)], associated with the IEM, were used to analyze envelope fractions. Polyclonal antibodies raised against ketol-acid reductoisomerase [anti-KARI from spinach, (Pontier et al., 2007)], a major polypeptide from the stroma, and the α, β, and γ subunits of the ATP synthase coupling factor 1 [anti-CF1 from spinach, (Pontier et al., 2007)], a major complex from thylakoids, were used to analyze stroma and thylakoid fractions, respectively. Immune complexes were detected using horseradish-peroxidase-conjugated anti-rabbit IgGs, and chemiluminescence visualization (ECL, Amersham Bioscience).

### Detection of biotinylated proteins

After electro-transfer of proteins separated by SDS-PAGE or 2-D PAGE on nitrocellulose, membranes were incubated overnight in 300 mM NaCl, 30 g·L^−1^ bovine serum albumine, 10 mM Tris-HCl, pH 7.5. Biotinylated proteins were detected on the blots after reaction with horseradish-peroxidase-conjugated streptavidin (Strep-HRP), and chemiluminescence visualization (ECL, Amersham Bioscience) according to the manufacturer instructions.

### Mass spectrometry and protein identification

After separation by 2-D PAGE, discrete spots were detected based on Coomassie blue-staining and excised from the gel. Correspondence between Coomassie-blue stained and chemiluminescent spots were determined based on comparisons using the ImageJ software (NIH). Relative quantities of proteins were assessed based on the staining intensity. Since Coomassie staining is not linearly correlated with absolute quantities of proteins, analyses were based on relative intensities, when comparing treated and untreated gels, allowing only most striking differences to be measured. An in-gel digestion was carried out as described (Ferro et al., 2000). Gel pieces were extracted with 5% [v/v] formic acid solution and acetonitrile from a gel corresponding to an untreated or treated sample. For this evaluation study, only one gel per condition was analyzed by mass spectrometry. Extracted peptides were desalted using C18-Zip Tips (Millipore). Elution of peptides was performed with 5–10 μl of a 50:50:0.1 (vol/vol) acetonitrile/H_2_O/formic acid solution. The tryptic peptide solution was introduced into a glass capillary (Protana, Odense, Denmark) for nanoelectrospray ionization. Tryptic peptide mass fingerprints were first assessed by matrix-assisted laser desorption/ionization time-of-flight mass spectrometry (MALDI-TOF/MS) analyses as described (Journet et al., 2000). Tandem mass spectrometry experiments were carried out on a Q-TOF hybrid mass spectrometer (Micromass). Interpretation of MS/MS spectra was achieved manually and with the help of the PEPSEQ program (MassLynx software, Micromass, Manchester, UK). MS/MS sequence information was used for database searching using the BLASTCOMP program (Ferro et al., 2002) performing BLAST searches for each amino acid sequence and clustering amino acid sequences identified from common BLAST hits. BLASTP and TBLASTN were used to mine plant protein and genomic databases, respectively.

## Results

### Selective superficial biotin-tagging and thermolysin-proteolysis of isolated chloroplasts

Superficial proteins from chloroplasts are potentially sensitive to non-permeable tagging or proteolysis. We sought therefore to “shave” or “tag” the surface of pure and intact chloroplasts isolated from spinach leaves.

“Shaving” was the easiest since treatment with the non-permeable protease thermolysin is a well-established method to digest proteins accessible at the outer surface of the OEM (Dorne et al., 1982; Joyard et al., 1983). Based on previous works, thermolysin appears therefore as a protease of choice, which cannot access the IEM or stroma. After proteolytic digestion of surface proteins, intact chloroplasts were re-isolated on a Percoll cushion, and chloroplast sub-compartment (envelope, stroma, thylakoids) were fractionated.

**Figure 1:**
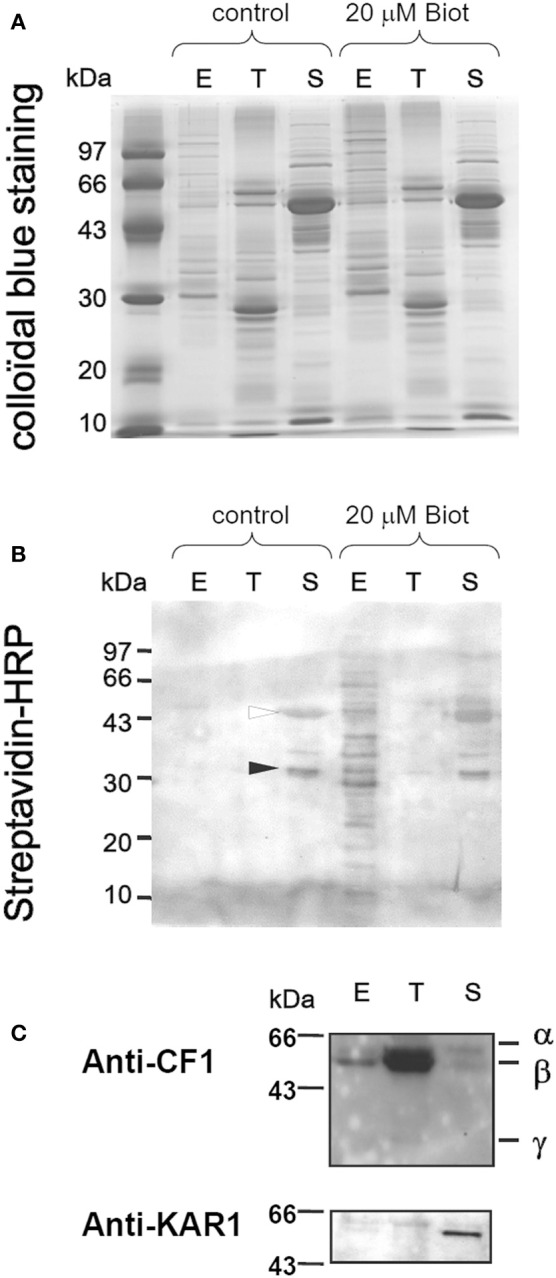
**Specific chloroplast envelope biotinylation**. Proteins prepared from intact (control) or biotinylated (20 μM Biot) spinach chloroplast sub-fractions (20 μg) were separated by 1D-PAGE. Polypeptides were either stained with Coomassie brilliant blue G- 250 **(A)** or electro-transferred to nitrocellulose membranes **(B,C)**. **(B)** Biotin was detected after reaction with streptavidin coupled to horse-radish peroxidase (HRP). Naturally biotinylated proteins from the stroma are indicated in control conditions (black and white arrows, see text). **(C)** Western blot detection of the CF1 ATPase α, β, and γ subunits from thylakoids (anti-CF1 1/10.000) and of the ketol-acid reductoisomerase from the stroma (anti-KARI 1/5000). E, envelope; T, thylakoids; S, stroma.

Superficial protein tagging with a biotinyl group was described for *Escherichia coli* (Bradburne et al., 1993) and *Helicobacter pylori* (Sabarth et al., 2002). For our purpose, we used the biotinylation reagent 6-((6-((biotinoyl)amino)hexanoyl)amino)hexanoic acid, succinimidyl ester (biotin-XX,SE). To prevent passive diffusion of the reagent through the OEM porine, (i) biotin-XX,SE (Mr 568) was selected for its relative hydrophobicity, (ii) biotinylation reaction was short (15 min), and (iii) temperature was kept low (4°C). By this mean, no biotinylation of IEM markers was detected. The succinimidyl ester reacts covalently with primary amino groups, i.e., the accessible *N*-termini and the ε-amino from lysyl residues. After biotinylation, intact chloroplasts were re-isolated on a Percoll gradient, and chloroplast sub-compartments (envelope, stroma, thylakoids) were fractionated. To control the efficiency of the biotinylation, after electrophoresis and transfer on nitrocellulose membranes, tagged proteins were visualized after reaction with streptavidin conjugated to horseradish peroxidase, and chemiluminescence detection. Figure 1A shows a 1-D SDS PAGE analysis of 20 μg proteins from envelope, thylakoid and stroma fractions obtained from chloroplast treated in the absence (left) or presence (right) of biotin-XX,SE. Figure 1B shows the biotin detection in each fraction following streptavidin reaction. Antibodies raised against ketol-acid reductoisomerase (anti-KARI), a major polypeptide from spinach chloroplast stroma, and the α, β, and γ subunits of the ATP synthase coupling factor 1 (anti-CF1), a major protein from thylakoids, were used to analyze stroma and thylakoid fractions, respectively, (Figure 1C). The α and β subunits were detected in both the envelope and stromal fractions, with a relative enrichment of the β subunit in the envelope and of the α subunit in the stroma (Figure 1C). As expected, naturally biotinylated stromal proteins could be detected (Figure 1B, control). In Figure 1B a black arrow indicates the band migrating at the molecular weight of biotin carboxyl carrier protein (BCCP), a subunit of the acetyl-CoA carboxylase complex (Alban et al., 1995; Elborough et al., 1996). Additional naturally biotinylated plastid proteins (Elborough et al., 1996) were also visualized, including a major band migrating at a molecular weight of ~50 kDa (Figure 1B, control) that might correspond to the unknown 50 kDa biotin-binding protein observed in rapeseed by Elborough et al. (1996). Only a few biotin-containing proteins have been characterized in plants, involved in the catalysis of carboxylation reaction or containing a non-catalytic biotin (Nikolau et al., 2003), including a geranoyl-CoA carboxylase protein, localized in the plastid in maize, and which gene sequence has to be determined. We did not characterize the streptavidine-binding proteins we observed in the stroma of spinach chloroplasts and do not propose any tentative identification for the corresponding bands. These results confirm that in our conditions, after treatment of isolated chloroplasts with the biotinylating reagent, the envelope fraction was the predominent compartment to be differentially tagged (Figure 1B, +20 μM biot).

Envelope proteins of untreated chloroplasts or after treatment with biotin-XX,SE (“tagged” chloroplasts) or with thermolysin (“shaved” chloroplasts) were subjected to 2-D PAGE (see below). Surface proteins might exhibit primary amino groups possibly tagged with biotin. They might also exhibit leucine or phenylalanine residues possibly cleaved by thermolysin. Alternatively, some surface proteins may not exhibit any such residues and be not altered by “tag” or “shave” treatments. Thus, Table 1 summarizes the simplest surface prediction of a protein according to its sensitivity to the “tag and shave” treatments. A single biotinylation induces a molecular weight increase of ~0.45 Da and no charge modification. As a result, 2-D PAGE resolution of biotinylated proteins should be nearly undistinguishable from the pattern of the corresponding non-biotinylated proteins.

**Table 1:** **Initial assessment of envelope peripheral protein topology after “tag” or “shave” positive reactions**.

**Biotinylation (tagged)**	**Thermolysin sensitivity (shaved)**	**Protein at the surface of the organelle**
−	−	?
+	−	yes
−	+	yes
+	+	yes

(?) In the absence of biotinylation or sensitivity to thermolysin, although the polypeptide is probably not exposed at the surface of the plastid envelope, one cannot exclude a localization at the OEM.

### Two-dimensional electrophoresis of chloroplast subfractions

**Figure 2:**
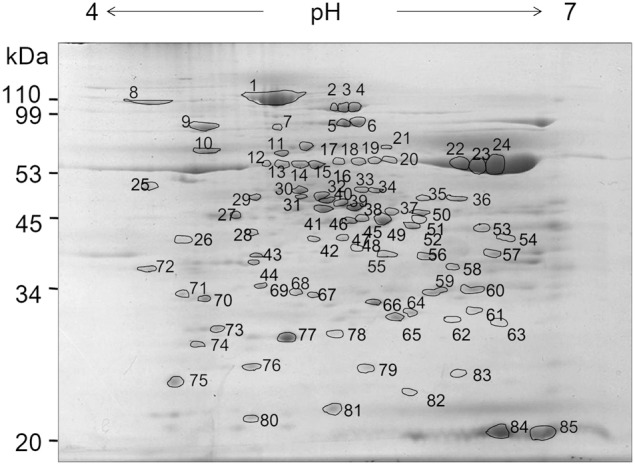
**Two-dimensional electrophoresis of spinach chloroplast envelope proteins**. Chloroplast envelope proteins (200 μg) were loaded onto the focusing gel (pH 4–7), resolved by 2-D PAGE and visualized by colloidal blue staining. A total of 85 protein spots from this map were excised, digested, and analyzed by MALDI-ToF to yield a peptide mass fingerprint for databases searching. Numbers indicate spot numbers. Apparent molecular weight (Mr) in kDa are indicated on the left.

We performed 2-D PAGE analyses of chloroplast sub-fractions based on methods previously developed to analyze thylakoid peripheral and lumenal proteins (Peltier et al., 2000; Schubert et al., 2002). We thus attempted to adapt the 2-D PAGE procedure for envelope samples in order to limit the poor yield in integral proteins as much as possible. Protein samples were loaded into the first electrophoretic dimension gel by rehydration of dried IEF strips (passive hydration of linear IPG strips). In most works, re-hydration of the IEF gel is usually carried out with an upper layer of mineral oil preventing water evaporation. By this technique, we noticed that after gel loading, Coomassie staining of the IEF rehydrated strip could detect little proteins, whereas substantial amounts of proteins were found in the mineral oil, with a 1-D SDS PAGE pattern close to that of chloroplast envelope (not shown). In addition to precipitation during IEF, membrane associated proteins were also lost by partition within the mineral oil before IEF loading. The 2-D PAGE resolutions reported here were therefore achieved after a 3-h passive hydration of linear IPG strips, without mineral oil over-layer, prior IEF and SDS 2-D PAGE. Figure 2 shows the envelope protein 2-D PAGE resolution after IEF on a 4–7 pH gradient. About 300 spots were visible after Coomassie staining, out of which 85 were circled for further gel comparisons. Some spots, such as 22, 23, and 24 trivially correspond to the Rubisco LSU. In results shown below, numbered spots in the vicinity of an immunostained-, tagged- or shaved spot were indicated.

**Figure 3:**
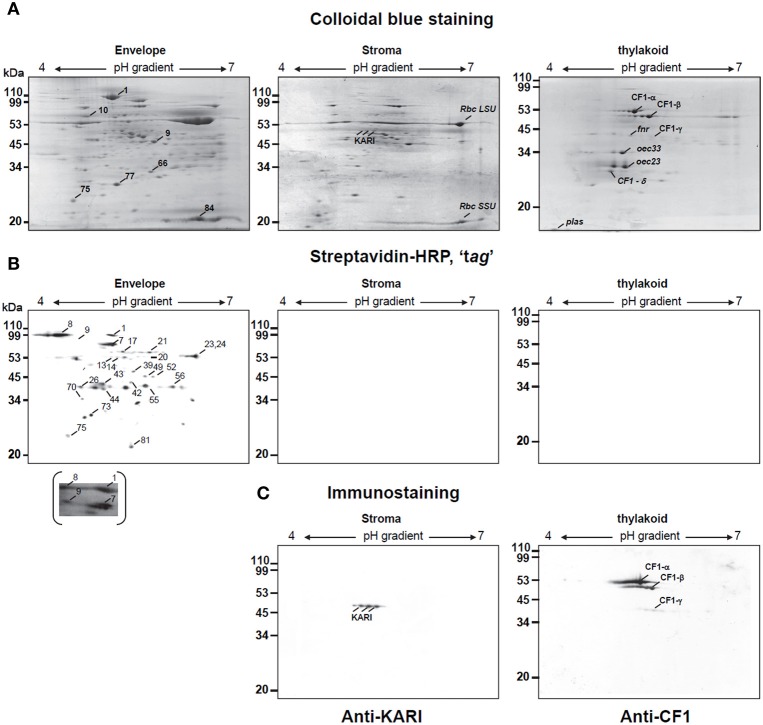
**Two-dimensional analysis of biotinylated proteins of envelope, thylakoid, and stroma from spinach chloroplast**. Chloroplast envelope (E), thylakoid (T), and stroma (S) proteins (200 μg) prepared from biotinylated spinach chloroplasts were passively loaded into 7 cm pH 4–7 IPG strips. Second-dimension separation was in 13% SDS polyacrylamide gels. Proteins were either stained with coomassie blue **(A)** or transferred to nitrocellulose membrane for biotine detection after Streptavidin Horseradish Peroxidase conjugated analysis (Streptavidin-HRP) **(B)** or for western-blotting using specific markers of stroma (anti-KARI) or thylakoids (anti-CF1) **(C)**. Major biotinylated spots were indicated on a representative gel **(C)**. In the case of spot 9, a longer exposure of the nitrocellulose membrane, revealing biotinylation is also shown. Rbc SSU, Rubisco small subunit; Rbc LSU, Rubisco large subunit; oec, oxygen evolving complex; plas, plastocyanin. Apparent molecular weight (Mr) in kDa are indicated on the left.

We then analyzed protein samples obtained after superficial tagging with biotin. Figure 3 shows the comparative resolution of proteins from envelope, stroma and thylakoids by 2-D PAGE after IEF on a 4–7 pH gradient. Protein patterns were accurately focused for further mass spectrometry analyses. Biotinylation of envelope proteins could be globally visualized after 2-D PAGE (Figure 3B, left). Antibodies raised against the stromal ketol-acid reductoisomerase (anti-KARI), and the thylakoid ATP synthase coupling factor 1 (anti-CF1, α, β, and γ-subunits), consistently reacted with polypeptides from the stroma and thylakoid fractions, respectively, (Figure 3C). Whereas immuno-cross-reactions were detected after 1-D PAGE of envelope proteins (see Figure 1C), immunostaining with anti-KARI or anti-CF1 was not detected after 2-D PAGE of envelope proteins (not shown). Similarly, the naturally biotinylated stromal proteins detected after 1-D PAGE (Figure 1B) were not detected after 2-D PAGE (Figure 3B, center). The chemiluminescence detection threshold of immunolabeled or tagged proteins is therefore lower after 2-D PAGE, probably reflecting the relatively lower yield of 2-D PAGE compared to 1-D PAGE, with a loss of parts of the hydrophobic proteins, a phenomenon we tried to control, but which should nevertheless be considered when analyzing results. An interesting consequence of this feature is that the immunostaining of a polypeptide resolved by 2-D PAGE indicates therefore a strong immunogenic reaction.

### Immunostaining-based proteomic assessment

**Figure 4:**
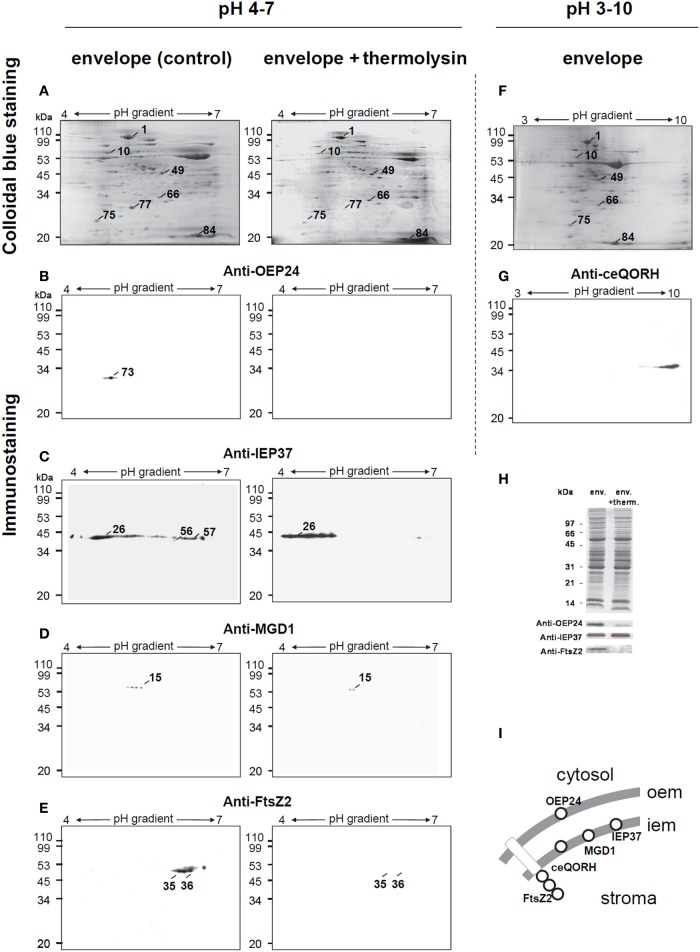
**Two-dimensional analysis of envelope proteins prepared from thermolysin treated intact spinach chloroplast. (A–D)** Isolated intact spinach chloroplasts were incubated in the absence (control) or presence (+ thermolysin) of the non-permeable protease thermolysin. After chloroplast disruption, envelope sub-fractions were recovered. Envelope proteins (200 μg) were passively loaded into 7 cm pH 4–7 IPG strips. Second- dimension separation was in 13% SDS polyacrylamide gels. Proteins were either stained with Coomassie blue **(A)** or transferred to nitrocellulose membrane for western-blotting using specific markers of the outer envelope membrane, anti-OEP24 **(B)** or the inner envelope membrane, anti-IEP37 **(C)**, anti-MGD1 **(D)** and anti-FtsZ2 **(E)**. **(F,G)** Spinach chloroplast envelope proteins (200 μg) were passively loaded into 7 cm pH 3–10 IPG strips. Second-dimension separation was in 13% polyacrylamide gels. Proteins were either stained with Coomassie blue **(F)** or transferred to nitrocellulose membrane for western-blotting using polyclonal antibodies against the recombinant Arabidopsis protein ceQORH **(G)**. **(H)** Standard SDS-PAGE analysis of spinach chloroplast envelope proteins used in the present study and western blotting using anti-IEP37, anti-0EP24, and anti-FtsZ2 polyclonal antibodies. **(I)** Schematic representation of protein markers. OEM, outer envelope membrane; IEM, inner envelope membrane. Apparent molecular weight (Mr) in kDa are indicated on the left.

Figure 4 shows the immunostaining-based identification of a set of envelope protein markers resolved by 2-D PAGE. Proteins were extracted from chloroplasts treated in the absence or presence of thermolysin, prior to sub-organellar fractionation (Figure 4A). Antibodies raised against OEP24, an OEM integral protein, IEP37, an IEM quinone methyl transferase and MGD1, the IEM MGDG synthase, reacted positively with envelope polypeptides (Figures 4B–D, left panels). The IEF of IEP37 was broad, an observed feature we could not explain. Main immunostaining was focused at the level of spot 26 (Figure 4C), in the acidic part of the gradient whereas the predicted IEP37 pI is 9.2 (Teyssier et al., 1996). Immunostaining at the level of spot 56 and to a lesser extent at the level of spot 57 possibly correspond to cross reactions of the antibody with minor proteins migrating at a slightly lower molecular weight compared to IEP37, with a higher pI and being sensitive to thermolysin treatment. Immunostaining of spinach chloroplast envelope proteins with the anti-IEP37 antibody can, in some cases, allow the detection of a broad band in 1D-PAGE (Figure 4H) possibly corresponding to some minor variations of the IEP37 sequence in the spinach leave samples we collected for our experiments. Two additional antibodies, raised against OEP10 and FtsZ2 associated to the OEM and IEM, respectively, also decorated envelope polypeptides (Table 2 and Figure 4E). Although OEP10 size is 6.7 kDa, the migration of this hydrophobic polypeptide containing one transmembrane segment in our 2-D PAGE was at the apparent molecular weight of 110, as assessed by both immunostaining and mass spectrometry determination (Table 2). As expected from their known localization in the OEM or IEM (Figure 4G), OEP24 and OEP10 were “shaved” away by the thermolysin treatment, whereas IEP37 and MGD1 were still detected in thermolysin-treated samples (Figures 4B–D, right panels). These analyses provide therefore a control for the accuracy of the thermolysin treatment. Interestingly, FtsZ2 immunostaining was not observed in envelope membranes purified from thermolysin-treated chloroplast, a phenomenon reported earlier (El-Kafafi et al., 2005) and consistent with: (i) the association of this peripheral protein of the IEM to an trans-envelope complex and (ii) the dependence of this association on the integrity of some of its OEM components.

**Table 2:** **Preliminary analysis of a selection of chloroplast-associated polypeptides, following biotinylation or thermolysin treatment and 2D-PAGE resolution**.

Polypeptides associated to the spinach envelope membranes based on immunostaining (*), MS- (**), or MS/MS (***) analyses	**Uniprot reference**	**Spot number**	**Mr (kDa)**		**Isoelectric point**		**Tag and shave**		**Protein localization (in Arabidopsis)**
			**Match**	**Gel**	**Match**	**Gel**	**Biotinylation**	**Thermolysin sensitivity**	
**STROMAL RNA EDITING**									
28RNP 28 kDa ribonucleoprotein (***)	P28644^a^	72	25.2	35.0	4.40	4.50	−	+++	Stroma^1^ + IEM^1^
CSP41 mRNA-binding protein (***)	O24365^a^	50	44.8	46.0	6.11	6.00	–	–	Stroma^1^ + Thyl^1^ + IEM^1^
**PROTEIN IMPORT COMPONENTS, CHAPERONES, AND PROTEASES**									
Cytosolic HSP70/Com70 (***)	P29357^a^	7	71.7	74.0	5.12	5.20	++	+++	Cytosol^2^ + OEM^2^
Translocon Tic110 (***)	Q8LPR9^b^	1	109.9	110.0	5.21	5,20	– (+ upper part of spot 1)	– (+ upper part of spot 1)	IEM^1^
Translocon Tic40-like protein (***)	Q9FMD5^b^	25	49.2	50.0	5.38	4.50	–	+++	IEM^1^
ClpC, member of the HSP100 family (**)	O98447^a^	2, 3, 4	99.4	99.0	8.78	5.50	–	+++	IEM^1^ + Stroma^1^
ClpP5, subunit of the ATP-dependent Clp protease (**)	Q9S834^b^	62	32.3	30.0	8.35	6.50	–	–	Stroma^1^ + IEM^1^
HSP70, member of the DNAK family (***)	O50036^a^	9	76.1	74.0	5.19	4.50	+	+++	Stroma^1^
Cpn21, member of the GroEL family, co-chaperone of Cpn60 (***)	Q02073^a^	60	26.9	35.0	8.45	6.50	–	–	Stroma^1^ + IEM^1^
Cpn60β, member of the GroES family, Rubisco binding-protein (***)	P08927^c^	17	62.9	60.0	5.85	5.30	– (+ on part of spot 84)	–	Stroma^1^
**RUBISCO, RUBISCO MATURATION AND ASSEMBLY**									
Rubisco LSU (**)	P00875^a^	23, 24	52.7	53.0	6.13	6.40	– (+ upper part of spot 23-24)	– (+ upper part spot 23-24)	Stroma^1^ + Env^1^
Rubisco SSU 2 (**)	Q43832^a^	84	20.3	21.0	8.24	6.50	–	–	Stroma^1^ + Env^1^
Rubisco SSU N-methyltransferase I (***)	Q9TIM3^a^	10	54.9	57.0	5.16	4.50	–	–	Stroma^1^
Rubisco activase (**)	P10871^a^	30, 38, 40	51.5	48–52.0	6.62	5.30	–	–	Stroma^1^
**OTHER CARBON METABOLISM COMPONENTS**									
Hexokinase 1 (***)	Q9SEK3^a^	20, 21	54.1	60.0	5.41	5.80	+++	+++	OEM^1^ + Mit./Vac./Nucl.^3^
Carbonic anhydrase (***)	P16016^a^	60	34.5	34.0	6.61	6.30	–	–	IEM^1^ + Stroma^1^
Phosphoglycerate kinase (***)	P29409^a^	54	45.5	42.0	5.83	6.50	–	–	Stroma^1^
Fructose-1,6-biphosphate aldolase (***)	P16096^a^/ P29356^a^	49	42.4	44.0	6.85	5.80	+	–	Stroma^1^/Cytosol^3^
Fructose-1,6-biphosphatase (***)	P22418^a^	55	37.1	37.0	5.52	5.50	+	–	Stroma^1^
Sedoheptulose-1,7-bisphosphatase (***)	O20252^a^	28	42.0	45.0	5.87	5.00	–	–	Stroma^1^
Transketolase (**)	O20250^a^	5, 6	80.2	75.0	6.20	5.50	–	–	Stroma^1^
Phosphoribulokinase (***)	P09559^a^	39	44.9	49	5.82	5.70	+	–	Stroma^1^
NAD-malate dehydrogenase (***)	O81609^c^	47	42.6	35.0	7.01	5.5	–	–	IEM^1^ + Stroma^1^
**THYLAKOID NUCLEAR ENCODED CF-1 SUBUNITS**									
CF1-ATP synthase alpha chain (**)	P06450^a^	11	55.4	57.0	5.16	5.20	–	–	Thyl^1^ + Env^1^
CF1-ATP synthase beta chain (**)	P00825^a^	16, 18	53.7	54.0	5.22	5.55	–	–	Thyl^1^
CF1-ATP synthase delta chain (**)	P11402^a^	74	27.6	28.0	5.80	4.50	–	–	Thyl^1^ + Env^1^
**ENVELOPE GLYCEROLIPID AND QUINONE METABOLISMS**									
MGD1 monogalactosyldiacylglycerol synthase (*)	Q9SM44^a^	12–15	53.7	54.0	5.22	5.00	–	–	IEM^1^
IEP37 quinone methyltransferase (*)	P23525^a^	~26	36.8	36.0	9.49	4–7	–	–	IEM^1^
CeQORH quinone oxydoreductase homolog (*)	Q9SV68^b^	nd	34.4	40.0	9.05	9.60	–	–	IEM^1^
**PLASTID DIVISION**									
FtsZ2 (*)	O82533^b^	nd	45.2	50.0	5.01	6.00	–	+++	IEM^4^ + Stroma^1^,^3^ + Thyl^3^
**OTHER ENVELOPE PROTEINS**									
OEP24 (*)	Q41393^a^	73	16.2	29.0	4.84	4.80	+++	+++	OEM^1^
OEP10 (* and ***)	P19407^a^	8	6.4	110.0	6.01	4.5	+++	+++	OEM^1^

The tag and shave intensity is given as a scale: −, no effect on the characterized spot; +, ++, +++, increasing effects of the tag or shave treatment. For biotinylation, the scale is estimated by the average size of the biotinylated spots being smaller, +; identical, ++; or bigger, +++; when compared to the size of the overlapping Coomassie-stained spots. (+), partial tag or shave of a large 2-D PAGE spot. UniProt accession numbers were given from

a Spinacea oleracea;

b Arabidopsis thaliana; and

c Pisum sativum. Abbreviations: Env, envelope; IEM, inner envelope membrane; Mit, mitochondria; OEM, outer envelope membrane; Thyl, thylakoïd; nd, not defined after Coomassie staining; Nucl, nucleus; Vac, vacuole. Previoulsy determined localization of proteins was obtained from works by

1 Ferro et al. (2010),

2 Ko et al. (1992),

3 Heazlewood et al. (2007), and

4 *El-Kafafi et al. (2005)*.

We sought whether the 2-D PAGE conditions we set up could accurately resolve basic membrane protein known to be particularly difficult to analyze by such technique. In pH 3–10 immobilized gradient conditions, polypeptides are detected after Coomassie staining in basic parts of the IEF pH gradient (Figure 4F). The ceQORH polypeptide, a basic IEM protein identified after organic solvent partition and 1-D SDS PAGE (Ferro et al., 2000; Miras et al., 2002), was immunodetected (Figure 4G). Together these data validate the quality of the 2-D PAGE conditions we used, based on the presence of envelope protein markers, consistently sensitive to the thermolysin superficial proteolysis, in a wide range of IEF pH gradient.

### Mass spectrometry-based proteomic assessment

Additional proteins were identified after mass spectrometry analyses. For this preliminary evaluation, we restricted or analysis to about 30 spots, for which a major protein could be identified following analysis. Indeed, although spinach chloroplast was the ideal starting material for this technical evaluation, the lack of genomic information on spinach was a clear limit of our study to identify proteins by mass spectrometry at a large scale and with a high resolution. Numerous spots allowed the detection of multiple proteins, which relative abundance could not be inferred and were not analyzed further. After discrete excision of spots, polypeptides were digested by trypsin inside the polyacrylamide gel and trypic fragments were subjected to MALDI-ToF to yield a peptide mass fingerprint for database searching. Sequence identification was further confirmed by MS/MS tryptic peptides analyses. Table 2 gives the list of proteins we assessed either by immunostaining or mass spectrometry analyses, with UniProt references of spinach proteins or corresponding homologs in *Arabidopsis* or pea in the absence of previously sequenced genes from spinach. Identified proteins include envelope membrane proteins and expectedly soluble proteins, either in the cytosol or chloroplast stroma. Stromal subunits of the CF-1 ATP-synthase, i.e., α, β, and γ subunits were further inventoried. Among soluble cytosolic and stromal proteins, 11 were previously known as envelope associated (Tranel et al., 1995; Rolland et al., 2003; El-Kafafi et al., 2005; Pontier et al., 2007; Ferro et al., 2010).

### Selective biotinylation and thermolysin-proteolysis of envelope peripheral proteins

**Figure 5:**
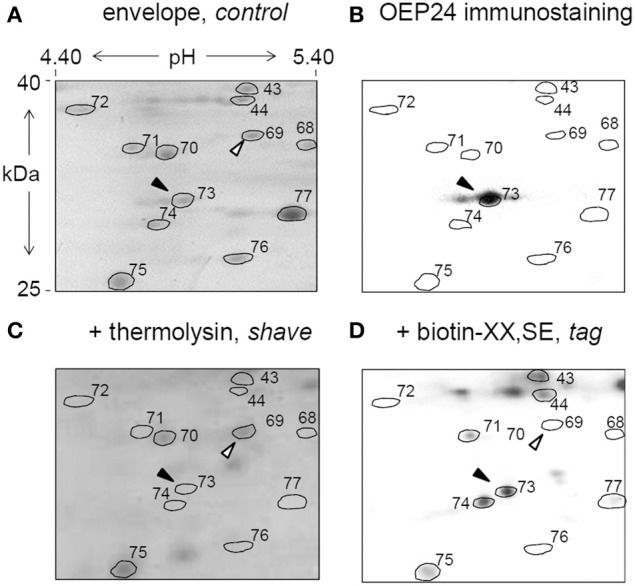
**Chloroplast envelope tag and shave**. Isolated chloroplasts were either untreated (control), treated with the non-permeable thermolysin protease (shave) or superficially biotinylated (tag), prior to envelope fractionation. Envelope proteins were subsequently resolved by 2D-PAGE as shown in Figure 2. Proteins were either stained with Coomassie blue **(A,C)** or transferred to a nitrocellulose membrane for Western-blotting using a specific markers of the outer envelope membrane, anti-OEP24 **(B)** or for biotin detection **(D)**. A magnified detail of the gel is shown. Spot 73 that corresponds to OEP24 (black arrow) is detected with the antibody raised against OEP24 **(B)**, and is consistently degraded (shaved) by the thermolysin protease **(C)** and biotinylated (tagged) by the biotin-XX,SE treatment. As a control, spot 69 (white arrow) is neither shaved nor tagged.

Table 2 collects information obtained from tag and shave experiments including proteins unaffected by neither of these treatments. This table includes proteins localized in the OEM or in other compartments of the chloroplast. It should not be considered as a comprehensive list, but rather as an evaluation of the tag and shave approach using the limited technique of 2D-PAGE. We noticed on few large spots (1, 23, 24), where the differential tagging or shaving was not homogenous, indicating that in these regions of the 2-D PAGE, more than one polypeptide was resolved in an apparent given spot. Figure 5 gives an example of a magnified area illustrating that an outer envelope protein, OEP24 (spot 73) was assessed by immunostaining (Figure 5B, solid arrow), was tagged (Figure 5D) and shaved (Figure 5C). As listed in Table 1, other patterns of differential tag or shave are noticed, such as spot 69 being neither tagged nor shaved (Figure 5, white arrow), as expected for a polypeptide that does not protrude at the surface of the chloroplast. In Table 2, cytosolic and superficial proteins are consistently tagged and/or shaved.

Surprisingly, although spots 2–4 (stromal protease ClpC) and spot 25 (translocon Tic40) are untagged by biotin, consistently with their association to the IEM, they disappear from the 2-D PAGE map of envelope prepared from thermolysin-treated chloroplasts. After a treatment by thermolysin or trypsin, it has been previously shown that, in whole protein extracts from chloroplasts, Tic40 was not degraded (Chou et al., 2003; Ko et al., 2005). The disappearance of spot 25 (Tic40) following thermolysin treatment is therefore puzzling. This phenomenon could not be attributed to an excessive proteolysis based on IEM protein markers, such as IEP37 and MGD1 that were still detected following thermolysin incubation. In pea (*Pisum sativum*), Tic40 has an apparent molecular weight of 44 kDa and has been previously detected in both IEM and OEM fractions (Ko et al., 1995), indicating a possible cohesive association with some OEM components; after incubation of pea chloroplast with thermolysin and analysis of envelope proteins, the treatment gave rise to a form with an apparent molecular weight of 42 kDa (Ko et al., 1995), which might explain that in our study, Tic40 might migrate to a position that differs from spot 25 of the 2-D PAGE. The cytosolic precursor of *Arabidopsis thaliana* Tic40 was also shown to be imported within chloroplasts in two steps, first as a soluble intermediate form, with an apparent molecular weight of 44 kDa and then as an IEM-associated form with an apparent molecular weight of 40.8 kDa (Li and Schnell, 2006). It is therefore also possible that the thermolysin treatment could affect the balance between an IEM-associated form and a soluble form of Tic40. Future works might help understanding the difference we observed. The parallel results observed for spots 2-4 (ClpC) and spot 25 (Tic40) is consistent with the co-immuno-precipitation of both Tic40 and ClpC with actin previously reported (Jouhet and Gray, 2009a,b; Franssen et al., 2011).

As mentioned above, a similar differential pattern was observed for FtsZ2 (Figure 4E). In the three cases we pointed here, the resolved spots match the mature Tic40, ClpC, or FtsZ2 proteins rather than their cytosolic precursors. These internal proteins are therefore inaccessible to thermolysin and their disappearance from the 2-D PAGE cannot be due to direct proteolysis. A possible common scenario could therefore be a disassembly from the IEM. Concerning FtsZ2, this division-ring component is indeed mostly present in the stroma of chloroplasts but also associated with the envelope as part of a trans-envelope complex that protrudes on the cytosolic side of the envelope (El-Kafafi et al., 2005; Falconet, 2012). Based on these three examples, which should nevertheless be confirmed by detailed analyses, in addition to OEM proteins detection, the tag and shave approach could also highlights IEM proteins which association to the IEM is strictly dependent on complexes protruding at the outer surface, and happens to be deeply destabilized when OEM components are cleaved by thermolysin-proteolysis.

## Discussion

### Topological and structural information brought by the “tag and shave” strategy and 2-D PAGE based proteomic analysis

It is still not known if an ideal electrophoretic technique would provide the exhaustive separation of both integral and peripheral envelope membrane-associated proteins, but the present study explores an optimized technique that might help completing the inventory initiated by 1-D PAGE based proteomic analyses. The proteins we identified after 2-D PAGE resolution were mainly peripheral or soluble. The confidence in results, concerning particularly the possible cross-contaminations, depends strongly on the purity of the treated material. For technical reasons, we used spinach chloroplasts as a working model, because of the high amount of starting material and the possibility to repeat experiments, but it is clearly not the ideal material since we lack some genomic information. Based on our evaluation, with molecular markers of spinach chloroplast sub-compartments and the exploration of the validity and limits of the method, this work can now serve as the basis for a well characterized model at the genomic and proteomic scales, such as *Arabidopsis thaliana*, pea (Franssen et al., 2011) or *Brassica rapa* (Cheng et al., 2011).

We thus paid attention to the sub-organellar fractionation methods and controlled the purity of the chloroplast sub-fractions using antibodies raised against stroma and thylakoid markers (Figures 1, 3). Some stromal proteins were associated, at least partly, to biotinylated spots, like the Fructose 1,6-bisphosphate aldolase or Cpn60-β (Table 2). The intensity of biotinylation in these two spots was low although the Coomassie staining was high. In the case of Fructose 1,6-bisphosphate aldolase, both cytosolic and stromal isoforms could be detected by mass spectrometry analyses (with marker peptides MVDVLIEQGIVPGIK and TVVSIPNGPSALAVK for the chloroplastic isoform; VTPEVIAEYTVR and TADGKPFVDAMK for the cytosolic one, Table 2). It is obvious that following this preliminary study, we need to determine whether polypeptides were tagged prior import, explaining the presence of biotinylated precursors of stromal proteins, or if multiple envelope proteins co-migrated at the same level of the 2-D PAGE, mixing polypeptides of various sub-compartments including biotinylated OEM proteins. Following this evaluation study, perspectives include the analysis of tagged/shave proteins with a method that does not depend on 2-D-PAGE. This is currently feasible using the high detection sensitivity of mass spectrometry applied to protein mixtures: search for biotin signatures in peptides analyzed by mass spectrometry will simply resolve this question.

**Figure 6:**
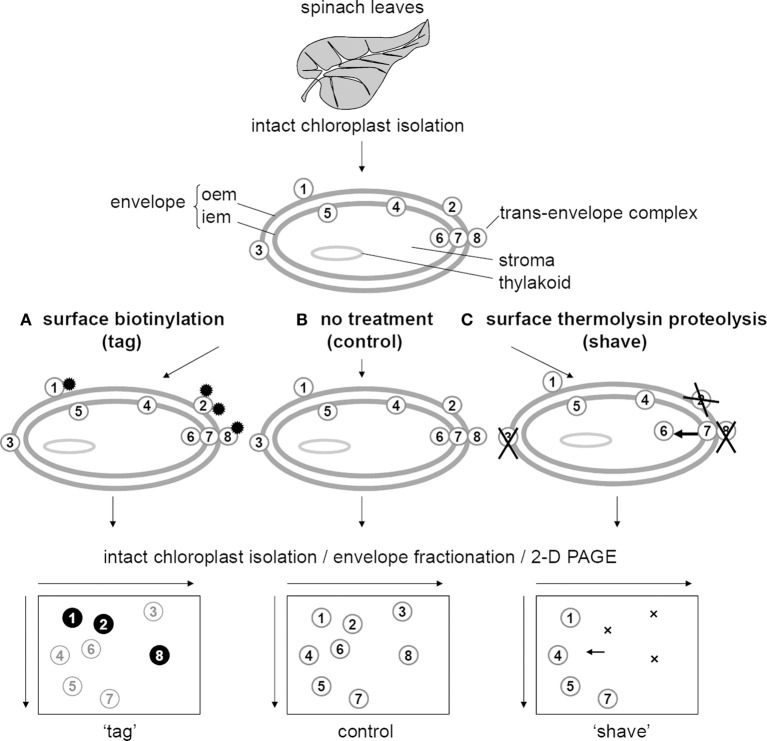
**Topological information possibly provided by the tag and shave strategy, coupled with 2D-PAGE proteomic analyses**. Chloroplasts isolated from leaves are either superficially tagged with biotin **(A)**, superficially shaved by proteolysis with thermolysin **(C)** or untreated **(B)**. **(A)** Outer envelope membrane (OEM) proteins that are possibly biotinylated are detected after biotin detection (proteins 1, 2, and 8), whereas OEM proteins that fail to be biotinylated are not tagged under these conditions (protein 3). Internal proteins (proteins 4, 5, 6, and 7) are not tagged. **(C)** Outer envelope membrane proteins that are possibly degraded by thermolysin disappear from the 2D-PAGE gel (proteins 2, 3, and 8, indicated by crosses), whereas OEM proteins that fail to be degraded are not shaved under these conditions (protein 1). Internal proteins are theoretically not shaved. However, internal proteins (such as protein 6) that bind to the IEM depending on the integrity of OEM proteins (stable trans-envelope complex, such as 6-7-8), will dissociate form the envelope after thermolysin treatment (protein 6, indicated by an arrow).

Motivation for an additional chloroplast envelope proteomic analysis should either be to provide information that is not available in other studies. The “tag and shave” strategy intended therefore to bring topological information, i.e., exposure of peripheral proteins at the surface of the organelle (Table 1, Figure 5). Insights on membrane-associated proteins involved in the sorting of cytosolic protein precursors, such as chaperones and translocon components, in the maturation and assembly of proteins, particularly Rubisco, in the carbon metabolism or in the stromal RNA editing, could therefore be obtained (Table 2). The present study also highlighted the disappearance from the envelope fraction of well-characterized internal proteins (Translocon Tic40 component, ClpC protease, FtsZ2), after treatment of intact chloroplasts by the non-permeable thermolysin protease (Table 2). The “tag and shave” strategy proved therefore to be informative on the stability of the association of these internal proteins to the IEM depending on the integrity of external superficial proteins associated to the OEM, shedding light on the importance of *trans-envelope* complexes stability. For example, FtsZ2 known to be involved in chloroplast division, has not been reported in proteomic analyses of pure envelope fractions from Arabidopsis (Ferro et al., 2010) although it binds to the IEM with a strength that depends on the integrity of some OEM proteins (El-Kafafi et al., 2005). FtsZ2 is therefore an obligate subunit of a stable trans-envelope complex. The results obtained with Tic40 would require a component of the inter-membrane space linking the IEM complex to the OEM, which presence and function should be demonstrated. Figure 6 summarizes all possible patterns after a “tag and shave” analysis. Trans-envelope complexes, which stability depends on the integrity of OEM components, are illustrated by the schematic spots 6, 7, and 8. Below, we discuss the most striking biological processes that appear to occur in the close vicinity of the chloroplast envelope membranes, involving proteins that associate in strong or lose complexes.

### General processes of cytosolic protein precursor import

Most chaperone, co-chaperones, proteases, and translocon subunits we identified were previously reported as major envelope-associated proteins (Rolland et al., 2003; Ferro et al., 2010). They are known to contribute to the general sorting processes of chloroplast protein precursors encoded in the nucleus. In the cytosolic side of chloroplasts, HSP70/Com70 molecular chaperones play a role in the handling of nucleus-encoded protein precursors (Zhang and Glaser, 2002; Salvucci, 2008; Vitlin et al., 2013). Cytosolic HSP70 proteins can specifically interact with targeting sequences of chloroplast precursors in a similar manner as mitochondrial precursors (Rial et al., 2000; Pontier et al., 2007). Chloroplast transit peptides differ from mitochondrial transit peptides by their phosphorylation on Ser and Thr residues in a phosphopeptide-binding motif for 14-3-3 proteins. The interaction of chloroplast precursors with the cytosolic HSP70 and 14-3-3 proteins was shown to enhance the translocation rate into chloroplasts (May and Soll, 2000). They make a “guidance complex” escorting the precursor to a specific TOC component (Li and Chiu, 2010; Lee et al., 2013). In this scenario, the guidance complex must be stable until it reaches OEM components. To our knowledge, no 14-3-3 protein was reported in envelope proteomic studies possibly due to its very dynamic destabilization and turnover or to the involvement of other escorting partners one might then find associated to the OEM (Flores-Perez and Jarvis, 2013). The presented strategy would then be the method of choice to identify these missing escorting proteins. The HSP70/Com70 protein we report here as a cytosolic protein partly associated to the chloroplast surface, might be the key protein that disrupts the guidance complex, once it reaches Toc34 (Lee et al., 2013). In the future, more sensitive analyses might also allow the detection of other components of the guidance complex.

The subsequent import of precursors implies the general translocon machinery, including several OEM (TOC) and IEM (TIC) subunits. Tic110 was shown to be the major component of the IEM import channel and is one of the most prominent proteins of the IEM (Soll, 2002). Tic110 is consistently found as a major protein in 2-D PAGE analyses performed here. On the stromal side of the IEM, Tic110 was also reported to be the binding site for molecular chaperones, including ClpC, a member of the HSP93-HSP100 family, and Cpn60. Here, both ClpC and Cpn60 were detected as stromal proteins associated to the IEM (Table 2). The dissociation of ClpC from the IEM occurred following treatment of chloroplasts with thermolysin (Table 2). This result indicates that the binding of ClpC depends on the integrity of proteins from the OEM. Tic40 also dissociated from the IEM when OEM proteins were subjected to proteolysis (Table 2). Tic40 is an IEM protein with a large hydrophilic domain in the stroma (Chou et al., 2003; Flores-Perez and Jarvis, 2013; Jarvis and Lopez-Juez, 2013). Cross-linking experiments showed that Tic40 is associated with Tic110, Toc75 and ClpC. The presence of Cpn60 could not be detected in Tic40 immunoprecipitates (Chou et al., 2003). Our study suggests that the strength of the association of Tic40-ClpC on the stromal side of Tic110 might depend on the integrity of protein components exposed at the outer surface of the chloroplasts. By contrast, Cpn60 association to Tic110 is not destabilized and involves therefore distinct binding mechanisms.

The initial association of protein precursors on the stromal side of the envelope is a fundamental event of protein import, because it brings a driving force for precursors through the translocon, bridging the TOC and TIC moieties, and because it prepares the accurate folding, processing, maturation and assembly on the stromal side. These processes involve general and specific chaperones, co-chaperones, co-factors and proteases (Li and Chiu, 2010; Lee et al., 2013). Here, we detect the stromal HSP70-DnaK homolog protein, ClpC, the ClpP1 subunit of the ClpP protease complex, the beta-subunit of Cpn60-GroEL and its co-chaperone Cpn21-GroES. All these proteins were previously reported in large-scale proteomic studies of chloroplast envelope membranes (Rolland et al., 2003; Ferro et al., 2010). ClpC is also known to direct specific proteins for degradation by the ClpP serine peptidase complex (Peltier et al., 2001), involved in the degradation of mistargeted or misfolded stroma proteins. In the turnover of TIC components, it is not known if ClpC could be involved in the degradation of some TIC proteins, like Tic40. The occurrence of both ClpC and a ClpP subunit encoded in the nucleus, i.e., ClpP5, at the periphery of the IEM, facilitates the route of some polypeptides toward ClpP degradation *via* ClpC. The ClpP5 subunit is still bound to the IEM after thermolysin treatment of chloroplasts (Table 2), whereas ClpC dissociates from the IEM under these conditions. Thus, although the ClpC chaperone and ClpP complex are topologically close and ready to interact, their association to the IEM is regulated differently *via* conformational status of other protein components.

The stromal Cpn60 chaperonin can form large tetradecamers, one containing the two stromal Cpn60 isoforms, i.e., Cpn60-α and Cpn60-β and the other consisting solely of Cpn60-β subunits (Dickson et al., 2000). The Cpn60-α/Cpn60-β tetradecamer is considered the major Cpn60 chaperonin in the stroma. Here, Cpn60-β is the sole subunit characterized in high amounts in the vicinity of the envelope and it is possible that a specific Cpn60-β tetradecamer might be associated to the IEM in spinach. The unique subunit that was detected in proteomic analyses of pure thylakoid membranes is the other isoform, Cpn60-α (Peltier et al., 2000). By contrast in *Arabidopsis*, only Cpn60-α could be found associated to the envelope (Ferro et al., 2010). The Cpn60-GroEL complex is known to form a central cavity that captures incompletely folded proteins. To that respect, Cpn21, a member of the GroEL family, was found associated to the envelope membranes (Table 2). This is consistent with the presence of a Cpn60/Cpn21 system that is functionally active in the IEM, in association with Tic110. Cpn60/Cpn21 is therefore topologically close to the imported protein precursors.

The chloroplast Cpn60 was initially identified as an abundant oligomeric protein that transiently binds the nascent large subunits of Rubisco, prior to their assembly into the Rubisco holoenzyme. Cpn60 chaperones are therefore often functionally annotated as a “Rubisco-binding protein.” The characterization of Rubisco SSU and LSU, of Cpn60 and its co-chaperonine Cpn21 and of Rubisco SSU N-methyltransferase in tight contact with the envelope membrane (Table 2) might be useful to better understand Rubisco SSU import, processing, methylation and assembly with LSU. The Rubisco SSU N-methyltransferase detected here (Table 2) has not been previously characterized in proteomic analyses of pure envelope membranes (Rolland et al., 2003; Ferro et al., 2010). An association to the IEM is supported by measures of O- and N-methylation of Rubisco SSU in purified envelope fraction, although such modification is not ubiquitous in the plant kingdom, apparently not essential and possibly minor in spinach (Mininno et al., 2012). Interestignly, the methyltransferase was also shown to be effective on another substrate, the fructose 1,6 bisphosphate aldolase (Mininno et al., 2012) also detected here (Table 2). This preliminary analysis also provided information that might be useful to better comprehend ATP synthase subunits import, assembly and possible association to envelope membranes (Table 2).

## Conclusion

In the present paper, we describe a method for a differential proteomic analysis of chloroplast envelope membrane peripheral proteins after 2D-PAGE resolution and immunological and mass spectrometry-based protein assessments. A basic proteomic snapshot of the most abundant proteins detected after Coomassie staining was investigated after treatment of intact chloroplasts following a superficial protein “tagging” with biotin or a superficial protein “shaving” with thermolysin. This evaluation study supports that information can be collected on the exposure of some OEM proteins at the surface of the chloroplast, but also on internal protein components, which association to the IEM relies on the stability of trans-envelope protein complexes and on the integrity of some OEM components. Future perspectives include an in-depth analysis of the envelope membrane proteome of “tagged or shaved” samples, using a more accurate plant model, such as Arabidopsis, with carefully purified chloroplasts. Perspectives include the analysis of the chloroplast envelope proteome using, more sensitive mass spectrometry analytical methods. The systematic analysis of biotinylated peptides by mass spectrometry (based on the mass shift introduced by biotin) will be a simple way to analyze the topology of OEM proteins, with possible cross contaminations by IEM or stromal precursors biotinylated in the course of their import. Following this evaluation of the method, the “tag and shave” strategy is therefore promising to bring refined topological information in large scale analyses. It could also be implemented, once validated, in the characterization of other membrane-limited organelles such as mitochondria.

### Conflict of interest statement

The authors declare that the research was conducted in the absence of any commercial or financial relationships that could be construed as a potential conflict of interest.
